# Different effects of catheter ablation on exercise tolerance, leg strength, and quality of life in paroxysmal versus persistent atrial fibrillation

**DOI:** 10.1002/joa3.13220

**Published:** 2025-01-14

**Authors:** Gen Matsuura, Hidehira Fukaya, Nobuaki Hamazaki, Daiki Saito, Hironori Nakamura, Naruya Ishizue, Tomoharu Yoshizawa, Jun Kishihara, Shinichi Niwano, Jun Oikawa, Junya Ako

**Affiliations:** ^1^ Department of Cardiovascular Medicine Kitasato University School of Medicine Sagamihara Japan; ^2^ Department of Rehabilitation Kitasato University Hospital Sagamihara Japan; ^3^ Department of Kitasato Clinical Research Center Kitasato University School of Medicine Sagamihara Japan

**Keywords:** a QOL questionnaire specific for AF, atrial fibrillation, catheter ablation, exercise tolerance, leg strength

## Abstract

**Background:**

Catheter ablation (CA) can improve exercise tolerance and quality of life (QOL) in patients with atrial fibrillation (AF). However, its differential effects on muscle strength between paroxysmal AF (PAF) and nonparoxysmal AF (Non‐PAF) remain unclear.

**Methods:**

We evaluated 94 patients (67.8 ± 10.3 years old, 71% male) who underwent CA (PAF/Non‐PAF 46/48) without AF recurrence. Six‐minute walk distance (6MWD), leg strength, and an AF‐specific QOL questionnaire (AFQLQ) were evaluated at baseline, 3, and 6 months after CA.

**Results:**

At baseline, the 6MWD and AFQLQ subset 3 score were significantly lower in patients with PAF than in those with Non‐PAF, but the parameters of muscle strength were comparable between the two groups. Both 6MWD and AFQLQ significantly improved at 6 months after CA in both groups. However, leg strength at 6 months after CA significantly improved in the Non‐PAF group (54.9 ± 16.5 to 58.4 ± 15.2, *p* < .05) but not in the PAF group.

**Conclusion:**

Successful CA for both PAF and Non‐PAF improved QOL and exercise tolerance. Additionally, CA improved leg strength in Non‐PAF patients.

## Graphical abstract

Successful catheter ablation (CA) can improve exercise tolerance and quality of life (QOL) in patients with atrial fibrillation (AF); however, its differential effects on muscle strength between paroxysmal AF (PAF) and nonparoxysmal AF (Non‐PAF) remain unclear. Successful CA improved QOL and exercise tolerance in both PAF and Non‐PAF patients, with additional improvements in leg strength observed in Non‐PAF patients.

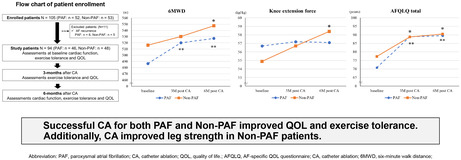

## 1 INTRODUCTION

Atrial fibrillation (AF) is the most common cardiac tachyarrhythmia, with an incidence and prevalence that increase with age.^1^, ^2^ AF is associated with a higher risk of severe complications, such as stroke, heart failure (HF), and death, along with various symptoms that negatively affect exercise tolerance, activities of daily living (ADL), and quality of life (QOL).^3^, ^4^, ^5^, ^6^

Catheter ablation (CA) is an established treatment for AF^1^ and may be more effective than antiarrhythmic drugs (AADs) for maintaining sinus rhythm (SR). Several studies have demonstrated that maintaining SR after successful CA improves outcomes, including reduced stroke rate, enhanced survival, and slower progression of HF.^7^, ^8^, ^9^, ^10^, ^11^ Furthermore, successful CA is well documented to improve the QOL and exercise tolerance, particularly in patients with persistent or long‐standing persistent AF.^12^, ^13^ In addition, maintaining SR after CA may enhance QOL and exercise tolerance in AF patients, including those without disabling symptoms, such as dyspnea on exertion.^14^, ^15^

Recent studies have reported that successful CA improves QOL and exercise tolerance in patients with both paroxysmal AF (PAF) and nonparoxysmal AF (Non‐PAF),^16^ regardless of AF type. However, data on the differential effects of CA on exercise tolerance, muscle strength, and QOL between PAF and Non‐PAF remain limited. Therefore, this study aimed to evaluate the different effects of successful CA on exercise tolerance, leg strength, and QOL in PAF and Non‐PAF patients.

## 2 METHODS

### 2.1 Study design

**FIGURE 1:**
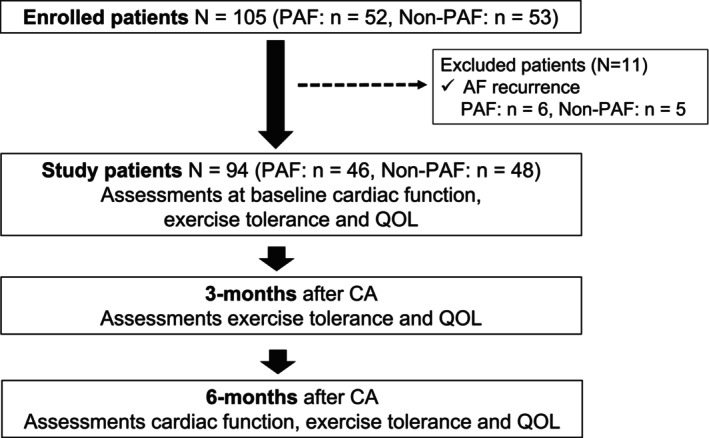
Flow chart of patient enrollment. CA, catheter ablation; PAF, paroxysmal atrial fibrillation; QOL, quality of life.

The present study complied with the Declaration of Helsinki and was approved by the institutional ethics committee of Kitasato University Hospital (approval number: B19‐217). All patients provided informed consent to participate in the study. This prospective, observational, single‐center study included consecutive patients (*n* = 105) who underwent initial CA for nonvalvular AF at our institute between March 2019 and February 2021. Eleven patients were excluded because of AF recurrence, leaving 94 patients (PAF, *n* = 46; Non‐PAF, *n* = 48) without recurrence for analysis. Six‐minute walk distance (6MWD), leg strength, and QOL were evaluated at baseline, 3 months, and 6 months after CA. The flow chart summarizing the study design and outcome is presented in Figure 1.

### 2.2 Catheter ablation procedure

Antiarrhythmic drugs (AADs) were discontinued at admission for CA. Oral anticoagulants were initiated at least 1 month before the procedure. CA was performed under general anesthesia with propofol, dexmedetomidine, and fentanyl. A multielectrode catheter (BeeAT; Japan Lifeline Co., Ltd., Tokyo, Japan) was inserted via the jugular vein and positioned in the coronary sinus. An intracardiac echocardiography catheter (ViewFlex, Abbott, St. Paul, MN, USA, or SOUNDSTAR, Biosense Webster, Irvine, CA, USA) was used to guide the transseptal puncture. After a single transseptal puncture, unfractionated heparin was intravenously administered to maintain an activated clotting time of >300 s. All patients underwent PVI by radiofrequency catheter ablation (RFCA) or cryoballoon ablation (CBA). In RFCA, extensive encircling PVI was performed using an electro‐anatomic mapping system (CARTO: Biosense Webster, Irvine, CA, USA, Inc., EnSite: Abbott, St. Paul, MN, USA or Rhythmia: Boston Scientific, Marlborough, MA, USA). Point‐by‐point PVI was performed with an open‐irrigated‐tip catheter (RFCA, SMARTTOUCH SF: Biosense Webster, Irvine, CA, USA; TactiCath SE: Abbott, St Paul, MN, USA: STABLEPOINT: Boston Scientific, Marlborough, MA, USA) with the following settings: power set to 30–40 W, target CF >5 g, and interlesion distance of 4 mm with a 4‐mm tag. The esophageal temperature (Esophastar; Japan Lifeline Co., Ltd., Tokyo, Japan) alarm was set at 40°C. The endpoint of the procedure was the completion of the PVI, verified using a circular catheter. For CBA, PVI was performed using a cryoballoon (CBA, Arctic Front Advance, Medtronic, Minneapolis, MN, USA) with an inner lumen mapping catheter (Achieve; Medtronic, Minneapolis, MN, USA) with a 150–240 sec application in the four pulmonary veins. The phrenic nerve was continuously paced during freezing in the right superior and inferior PVs to evaluate phrenic nerve palsy. Additional ablation beyond PVI was left to the discretion of the operators.

### 2.3 QOL measurement

The QOL was assessed using an AF‐specific QOL questionnaire (AFQLQ),^17^, ^18^ which is valid and reproducible. AFQLQ consists of three subsets: (1) variety and frequency of symptoms (Questions 1–6; 0–24 points); (2) severity of symptoms (Questions 7–12; 0–18 points); and (3) limitations of daily and special activities as well as mental anxiety related to AF (Questions 13–26; 0–56 points). A higher score indicates better health status. In addition, the SARC‐F questionnaire was used to evaluate the sarcopenia. The SARC‐F consists of 5‐item self‐reported test addressing Strength, Assistance in walking, Rise from a chair, Climb stairs, and Falls (each 0–2 points).^19^ A score ≥4 indicates a higher risk of sarcopenia.^20^ Physical activity (PA) was assessed using a three‐question (3Q) assessment.

### 2.4 Exercise tolerance measurement

Exercise tolerance was assessed using the 6MWD test at baseline, 3 and 6 months after CA.^21^, ^22^ Patients were instructed to walk as fast and for as long as possible, adjusting their walking pace if they experienced palpitations, fatigue, or dyspnea. Handgrip strength was measured for each hand using a standard digital dynamometer (TKK 5101 Grip‐D, Takei, Tokyo, Japan). The average maximal value was used as the parameter for handgrip strength (kg). To evaluate a parameter of leg strength, maximal knee extensor strength was assessed for each leg using a hand‐held dynamometer (μTas F‐1, Anima, Tokyo, Japan) with patients sitting on a chair with their hips and knees flexed at a 90° angle. The average maximal value for both legs, divided by body weight (kg), was used as the knee extensor strength parameter. In addition, a multifrequency bioelectrical impedance analyzer (BIA), the InBody 470 (InBody Japan, Tokyo, Japan), was used according to the manufacturer's guidelines. The appendicular skeletal mass index (SMI) was determined as the sum of arm and leg lean mass (kg) divided by height squared (m^2^).

### 2.5 Follow‐up after CA

Anticoagulation therapy was continued for at least 3 months after CA. The decision to continue AADs until the end of the blanking period was at the discretion of each physician. All patients were followed up at 1, 3, and 6 months after CA. Laboratory data, echocardiography, and Holter ECG were performed at baseline and 6 months after CA. AF recurrence was defined as AF episodes lasting more than 30 s after the 3‐month blanking period. Heart failure was defined as a history of HF or recent HF exacerbation (i.e., active within the past 100 days), and HF was determined based on the presence of HF symptoms, such as dyspnea and edema or the initiation of HF‐specific pharmacologic treatment. Successful ablation was defined as freedom from AF or atrial tachyarrhythmia during 6‐month follow‐up.

### 2.6 Statistical analysis

All statistical analyses were performed using JMP 14 software (SAS Institute Inc., Cary, NC, USA). Patient characteristics data are presented as the mean ± standard deviation or median values (25th and 75th interquartile range) for continuous variables and as the numbers and percentages for categorical variables. Differences between the two groups were analyzed using the chi‐squared test, the Wilcoxon rank‐sum test, or the *t*‐test. Repeated measures were used to compare exercise tolerance, leg strength, and QOL scores between baseline and follow‐up at 3 and 6 months after CA. The effect size was calculated using Hedges' g, a method for estimating the standardized mean difference. A paired *t*‐test was used to compare laboratory data and echocardiography results between baseline and follow‐up 6 months after CA. The level of statistical significance was set at *p* < .05.

## 3 RESULTS

### 3.1 Clinical characteristics of the study patients

Figure 1 shows the flow chart of patient enrollment. A total of 105 patients were initially enrolled in this study, and 11 patients were excluded because of AF recurrence. Therefore, the final study group included 94 patients (PAF, *n* = 46; Non‐PAF, *n* = 48).

Table 1 shows the baseline clinical characteristics of all patients, as well as those of the PAF and Non‐PAF groups. The mean age was 67.8 ± 10.3 years, and 67 patients (71.3%) were male. The HANP and BNP values were significantly lower in the PAF group than in the Non‐PAF group. The average LA dimension (LAD) was significantly smaller in the PAF group compared with the Non‐PAF group, but the left ventricular ejection fraction (LVEF) was comparable between the two groups. Regarding the AADs, class I AADs were more frequently administrated in the PAF group than in the Non‐PAF group, but the use of other types AADs was comparable between the two groups. RFCA for PVI and superior vena cava isolation (SVC‐I) were more frequently performed in the Non‐PAF group than in the PAF group. All patients successfully achieved PVI, and no major complications occurred during or after the procedure.

**TABLE 1:** Patient characteristics.

	All (*n* = 94)	PAF (*n* = 46)	Non‐PAF (*n* = 48)	*p*
Clinical characteristics				
Age (years)	67.8 ± 10.3	68.8 ± 10.5	66.8 ± 10.2	.3695
Gender, males, *n* (%)	67 (71.3)	36 (78.3)	31 (64.6)	.1410
BMI (kg/m^2^)	24.0 ± 3.7	23.9 ± 2.9	24.1 ± 4.4	.8296
Hypertension, *n* (%)	53 (56.4)	23 (50.0)	30 (62.5)	.2213
Diabetes mellitus, *n* (%)	7 (7.4)	4 (8.7)	3 (6.3)	.6513
Heart failure, *n* (%)	17 (18.1)	10 (21.7)	7 (14.6)	.3668
History of stroke, *n* (%)	8 (8.5)	4 (8.7)	4 (8.3)	.9498
CHADS_2_ score	1 [1, 2]	1 [0, 2]	1 [1, 2]	.5250
Laboratory data				
BNP (pg/mL)	109.1 [47.9, 202.4]	72.4 [34.0, 167.5]	168.0 [74.8, 218.8]	.0031
HANP (pg/mL)	91.8 [45.5, 134.5]	66.8 [37.5, 135.0]	103.0 [71.1, 136.5]	.0409
Cr (mg/dL)	0.9 [0.8, 1.0]	0.9 [0.8, 1.0]	0.9 [0.8, 1.0]	.6228
Echocardiographic parameter				
LVEF (%)	64.4 ± 7.2	65.0 ± 7.9	63.8 ± 6.3	.4163
LAD (mm)	40.0 ± 5.2	38.9 ± 5.5	41.1 ± 4.7	.0444
LVDD (mm)	47.7 ± 5.1	48.3 ± 5.9	47.1 ± 4.3	.2627
E/e’	8.2 ± 2.6	8.2 ± 2.8	8.2 ± 4.3	.9420
Medications				
Class I AAD, *n* (%)	14 (14.9)	11 (23.9)	3 (6.3)	.0137
Class II AAD, *n* (%)	42 (44.7)	21 (45.7)	21 (43.8)	.8529
Class III AAD, *n* (%)	8 (8.5)	4 (8.7)	4 (8.3)	.9498
Class IV AAD, *n* (%)	10 (10.6)	5 (10.9)	5 (10.4)	.9433
RAS‐I, *n* (%)	34 (36.2)	16 (34.8)	18 (37.5)	.7840
MRA, *n* (%)	5 (5.3)	3 (6.5)	2 (4.2)	.6102
Statin, *n* (%)	37 (39.4)	22 (47.8)	15 (31.3)	.0993
Ablation procedure				
RFCA‐PVI, *n* (%)	77 (81.9)	34 (73.9)	43 (89.6)	.0460
Posterior wall isolation, *n* (%)	5 (5.3)	3 (6.5)	2 (4.2)	.6102
SVC isolation, *n* (%)	24 (25.5)	6 (13.0)	18 (37.5)	.0056
Mitral‐isthmus ablation, *n* (%)	3 (3.2)	2 (4.3)	1 (2.1)	.5291
CTI ablation, *n* (%)	83 (88.3)	40 (87.0)	43 (89.6)	.6920

*Note*: Data presented as mean ± SD, median (25th, 75th interquartile range: IQR), or *n* (%).

Abbreviations: AAD, antiarrhythmic drug; BMI, body mass index; BNP, B‐type natriuretic peptide; Cr, creatine; CTI, cavo‐tricuspid isthmus; HANP, human atrial natriuretic peptide; LAD, left atrial dimension; LVDD, left ventricular end‐diastolic diameter; LVEF, left ventricular ejection fraction; MRA, mineralocorticoid receptor antagonist; PAF, paroxysmal AF; PVI, pulmonary vein isolation; RAS‐I, renin‐angiotensin system inhibitors; RFCA, radiofrequency catheter ablation; SVC, superior vena cava.

Table 2 presents the baseline exercise capacity and QOL questionnaire results for all patients, as well as for both groups. The 6MWD, maximal heart rate (HR) at 6MWD, and AFQLQ subset 3 scores at baseline were significantly lower in the PAF group than in the Non‐PAF group. However, the parameters of muscle strength, SMI, AFQLQ subset 1, 2, and SARC‐F scores were comparable between the two groups.

**TABLE 2:** Exercise tolerance parameters and QOL questionnaire tests at baseline.

	All (*n* = 94)	PAF (*n* = 46)	Non‐PAF (*n* = 48)	*p*
Exercise tolerance parameters				
6MWD (m)	504.1 ± 82.2	486.6 ± 74.1	521.2 ± 86.8	.0418
Maximal HR at 6MWD (bpm)	126.9 ± 29.3	113.8 ± 29.9	139.8 ± 29.9	< .0001
Mean grip strength (kg)	32.5 ± 9.6	32.6 ± 8.9	32.4 ± 10.2	.9240
Mean knee extension force (kgf/kg)	55.8 ± 14.5	56.7 ± 12.2	54.9 ± 16.5	.5514
SMI [(kg)/height (m)]^2^	7.2 ± 1.0	7.0 ± 0.2	7.3 ± 0.2	.2794
QOL score				
AFQLQ score				
Total score (points)	74.1 ± 17.0	70.8 ± 17.8	77.3 ± 15.8	.0674
AFQLQ1 (points)	15.9 ± 6.8	15.0 ± 7.0	16.9 ± 6.6	.1883
AFQLQ2 (points)	13.7 ± 4.4	13.2 ± 4.6	14.2 ± 4.2	.2807
AFQLQ3 (points)	44.4 ± 8.2	42.6 ± 8.8	46.2 ± 7.2	.0339
SARC‐F (points)	0.8 ± 1.2	0.8 ± 1.1	0.8 ± 1.2	.6214

*Note*: Data presented as the mean ± SD.

Abbreviations: 6MWD, 6‐minute walk distance; AFQLQ, atrial fibrillation quality of life questionnaire; HR, heart rate; QOL, quality of life; SMI, skeletal mass index.

### 3.2 Changes in QOL after the CA

**FIGURE 2:**
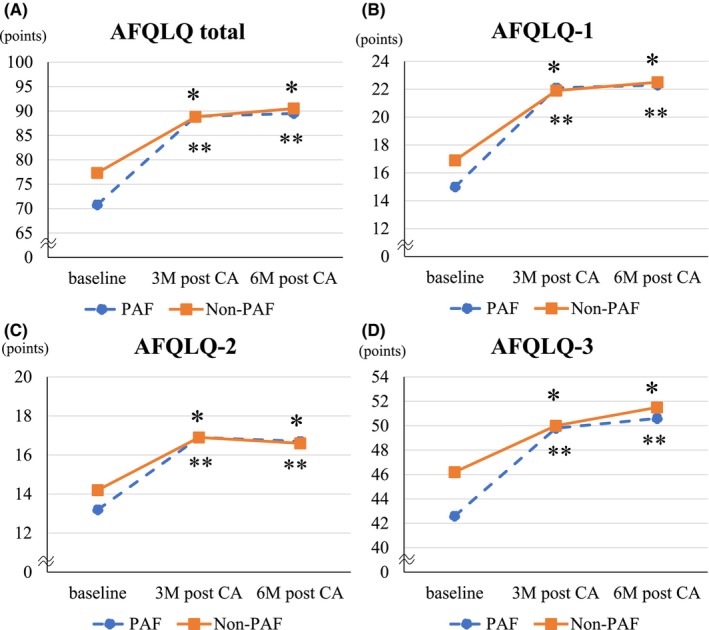
Changes in AFQLQ score [(A) AFQLQ‐total, (B) AFQLQ‐1, (C) AFQLQ‐2, (D) AFQLQ‐3] from baseline to follow‐up. AFQLQ scores at 3 and 6 months after CA significantly improved compared with those at baseline in all subsets in the PAF and Non‐PAF groups. The effect size measured by Hedges' g showed a large effect in both groups (Hedges' g of PAF/Non‐PAF groups = 1.7/1.3 at 3 M after CA, 1.8/1.5 at 6 M after CA). AFQLQ, AF‐specific QOL questionnaire; CA, catheter ablation; PAF, paroxysmal atrial fibrillation. *p < .05 versus Compared with Baseline in the Non‐PAF group, **p < .05 versus Compared with Baseline in the PAF group.

Figure 2 shows changes in AFQLQ scores from baseline to follow‐up. In both PAF and Non‐PAF groups, AFQLQ scores significantly improved at 3 and 6 months after CA compared with baseline in all subsets. The effect size measured by Hedges' g showed a large effect in both groups (Hedges' g of PAF/Non‐PAF groups = 1.7/1.3 at 3 M after CA, 1.8/1.5 at 6 M after CA). Although the scores were significantly improved at 3 months, nor further improvement was observed beyond this point during the follow‐up period in either group. The SARC‐F at 6 months after CA significantly improved in the Non‐PAF group (Hedges' g = 0.9) but not in the PAF group. The 3Q assessment at 3, and 6 months after CA significantly improved in the Non‐PAF group (Hedges' g = 0.5 at 3 M after CA, 0.6 at 6 M after CA) but not in the PAF group (Table S1).

### 3.3 Changes in exercise tolerance after CA

**FIGURE 3:**
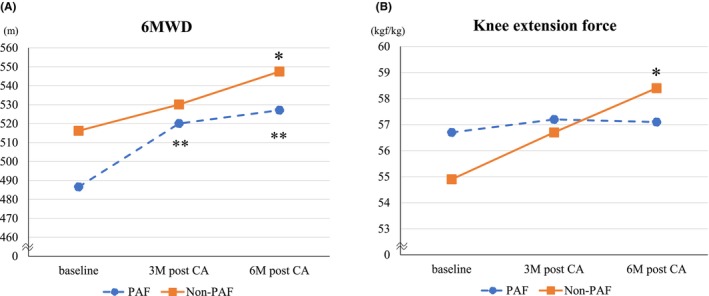
Changes in (A) 6MWD and (B) Knee extension force from baseline to follow‐up. The 6MWD at 6 months after CA significantly improved compared with those at baseline in both groups (Hedges' g of PAF/Non‐PAF groups = 1.1/1.1). The leg strength at 6 months after CA significantly improved compared with those at baseline in the Non‐PAF (Hedges' g = 0.5) but not in PAF group. 6MWD, 6‐minute walk distance; CA, catheter ablation; PAF, paroxysmal atrial fibrillation. *p < .05 versus Compared with Baseline in the Non‐PAF group, **p < .05 versus Compared with Baseline in the PAF group.

Baseline exercise tolerance data are presented in Table 2. Patients in the Non‐PAF group had higher 6MWD and a maximal HR at 6MWD than those in the PAF group, but mean grip strength, mean knee extension force, and SMI were comparable between the two groups. Figure 3 shows changes in exercise tolerance from baseline to 3 and 6 months. The 6MWD at 6 months after CA significantly improved compared with baseline in both groups (Hedges' g of PAF/Non‐PAF groups = 1.1/1.1). In the Non‐PAF group, 6MWD gradually improved over the 6‐month follow‐up period, whereas in the PAF group, improvement was observed at 3 months but did not significantly change thereafter. Maximum HR at 6MWD significantly improved at 6 months after CA in the Non‐PAF (Hedges' g = 1.5) but not in the PAF group. Leg strength also significantly improved at 6 months after CA in the Non‐PAF (Hedges' g = 0.5) but not in the PAF group. Moreover, body weight reduced at 3 and 6 months after CA in the Non‐PAF (Hedges' g = 0.5 at 3 M after CA, 0.5 at 6 M after CA) but not in the PAF group. In both PAF and Non‐PAF groups, mean grip strength and SMI did not show significant differences before and after the procedure (Table S1).

### 3.4 Effects of sinus rhythm maintenance on other parameters

**FIGURE 4:**
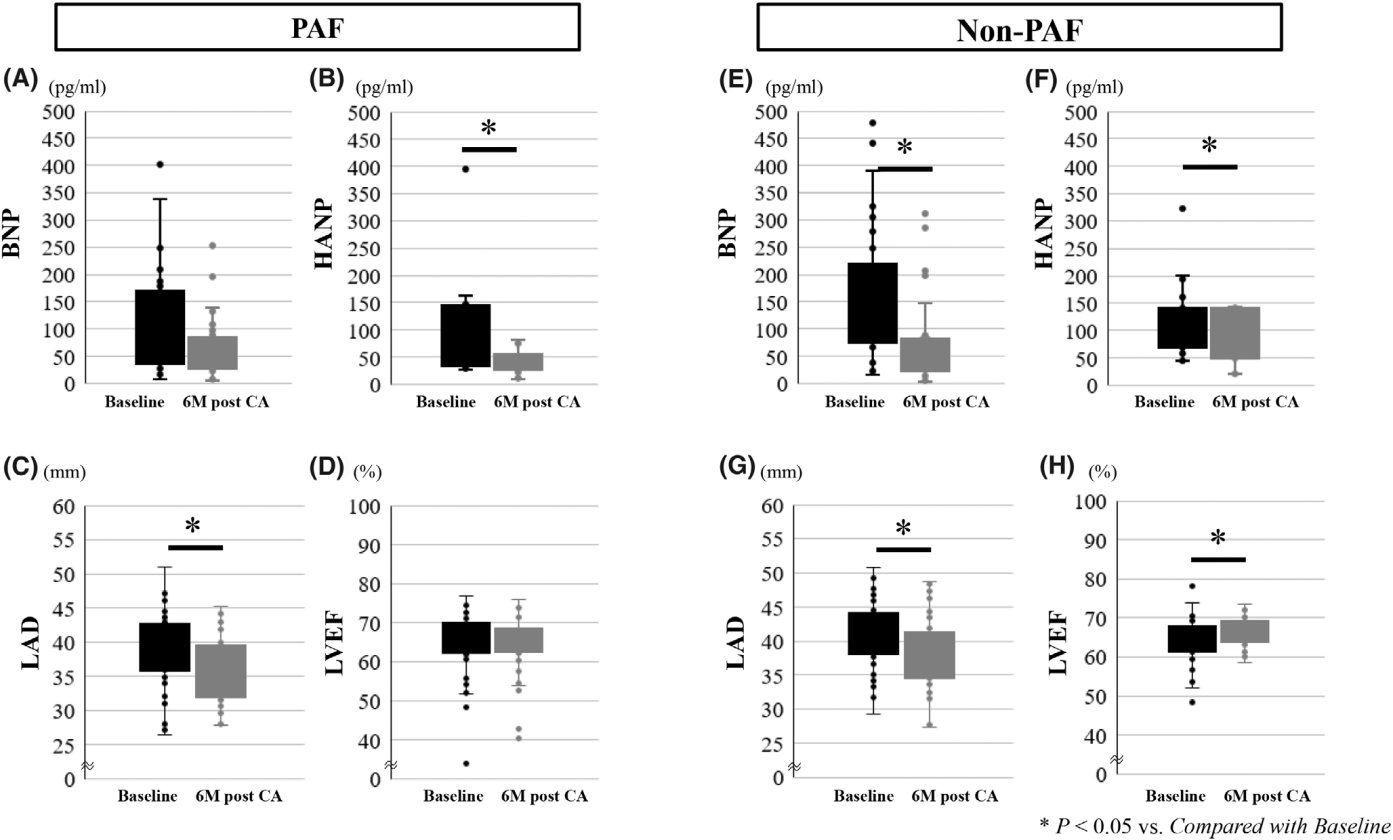
Changes in BNP, HANP, LAD, and LVEF from baseline to follow‐up. The BNP (A and E) and LVEF (D and H) at 6 months after CA significantly improved compared with those at baseline in the Non‐PAF but not in PAF group. The HANP (B and F) and LAD (C and G) at 6 months after CA significantly improved compared with those at baseline in both groups. BNP, B‐type natriuretic peptide; CA, catheter ablation; HANP human atrial natriuretic peptide; LAD, left atrial dimension; LVEF, left ventricular ejection fraction; PAF, paroxysmal atrial fibrillation.

Figure 4 shows changes in BNP, HANP, LAD, and LVEF from baseline to 3 and 6 months of follow‐up. BNP and LVEF significantly improved at 6 months after CA compared with baseline in the Non‐PAF group, but not in the PAF group. HANP and LAD significantly improved at 6 months after CA in both groups compared with those at baseline (Table S2).

## 4 DISCUSSION

### 4.1 Main finding

In this study, we evaluated the impact of successful CA on exercise tolerance, leg strength, and QOL in patients with PAF and Non‐PAF. Our findings are as follows: (1) successful CA improved QOL and exercise tolerance regardless of AF type; (2) CA also improved leg strength in Non‐PAF patients.

### 4.2 The impact of successful CA on QOL and exercise tolerance in patients with AF

AF is associated with various symptoms that negatively affect exercise tolerance, ADL, and QOL.^3^, ^4^, ^5^, ^6^ Impaired QOL and exercise tolerance may be explained by several mechanisms, including the loss of the atrial contribution to left ventricular filling, irregular RR intervals, valvular regurgitation, inappropriate increases in HR, or reduced cardiac output.^23^, ^24^ Several studies have demonstrated that successful CA improved the QOL and exercise tolerance in symptomatic and asymptomatic AF patients.^14^, ^15^ Additionally, Mohanty et al. reported^15^ that successful CA significantly reduced resting and peak HR and increased peak oxygen pulse, while no improvement was observed in patients with failed CA. These findings suggest that maintenance of SR via CA could be associated with improvements in exercise tolerance, which may be impaired because of AF. In our study, in both PAF and Non‐PAF groups, 6MWD and AFQLQ scores significantly improved after CA compared with baseline. Furthermore, the physical activity using the 3Q assessment improved at 3 and 6 months after CA in the Non‐PAF but not in the PAF group. Interestingly, leg strength at 6 months after CA significantly improved in the Non‐PAF but not in the PAF group. Therefore, we can assume that CA also improved leg strength in Non‐PAF patients because of gain in daily activity after CA. The influence of PAF on exercise tolerance remains unclear; however, it may involve different mechanisms compared with Non‐PAF.

### 4.3 Different effects of CA on the exercise tolerance and QOL in PAF vs. persistent AF

In the present study, we evaluated the impact of successful CA on exercise tolerance, leg strength, and QOL in different types of AF: PAF and Non‐PAF. The improvement in QOL was consistent with the maintenance of SR by PVI, as reported in several studies.^15^ Our study demonstrated that AFQLQ scores improved at 3 months after CA and remained stable without further changes during the follow‐up period in both PAF and Non‐PAF groups. These findings suggest that achieving durable PVI can lead to a relatively quick improvement in QOL, regardless of the type of AF. Recently, it was reported that successful CA also improved exercise tolerance in patients with PAF and Non‐PAF, regardless of the type of AF. Mujović NM et al. demonstrated^16^ a significant improvement in the respiratory exchange ratio (RER) at maximal workload in patients with PAF following CA. Several studies have shown that the loss of effective LA mechanical function occurred in patients with PAF.^25^, ^26^, ^27^ Our study also showed that HANP and LAD significantly improved at 6 months after CA in both PAF and Non‐PAF groups. Furthermore, the changes in QOL after CA that affect physical activity may differ between PAF and Non‐PAF. Kato et al. reported^28^ that the effects of restoring SR following CA on the changes in QOL differed between patients with PAF and Non‐PAF; Mental QOL improved only in patients with PAF following PVI, while physical QOL improvement was greater in Non‐PAF patients. Our study also showed that the AFQLQ Subset 3 score, which reflected limitations of daily and special activities as well as mental anxiety related to AF, was significantly lower in the PAF group than in the Non‐PAF group before CA. Most PAF patients had symptoms related to AF is intermittent, and the disappearance of these symptoms after CA might have stabilized their mental status. As a result, physical activity might have improved. However, no improvement in leg strength and muscle mass was observed in the PAF groups, indicating that the mechanism of exercise tolerance improvement due to increased physical activity might differ in PAF patients. There may be undetectable pathophysiological processes behind the causes associated with impaired exercise tolerance in patients with PAF, and future studies are needed to elucidate the detailed mechanisms.

### 4.4 Clinical implications

Successful CA can improve exercise tolerance and QOL in patients with AF. However, few studies have compared the effects of CA between PAF and Non‐PAF, particularly regarding muscle strength. In this study, we evaluated the differential effects of successful CA on exercise tolerance, leg strength, and QOL in PAF and Non‐PAF patients. Our results demonstrated that successful CA improved QOL and exercise tolerance in both PAF and Non‐PAF patients, with additional improvements in leg strength observed in Non‐PAF patients. Therefore, the mechanism of physical performance improvement might differ in PAF and Non‐PAF patients. These findings provide valuable insights for clinicians when considering CA for improving QOL and ADL, and muscle strength in AF patients by the CA.

### 4.5 Study limitations

This study has several limitations. First, this study was a single‐center study with a relatively small sample size. In addition, it was a non‐randomized study without a control group, which could introduce selection bias and unmeasured confounders. Second, we did not perform follow‐up exercise tolerance tests or QOL assessments with AF recurrence; therefore, the influence of CA on improving those parameters in the presence of recurrence remains unclear. Third, exercise tolerance tests and QOL tests were evaluated only at 3 and 6 months after CA, with no data available from the chronic phase due to the COVID‐19 pandemic. As a result, future studies are needed to examine the long‐term effects of SR maintenance after CA. Finally, observed improvements in exercise capacity and QOL might have been influenced by multiple other factors. Additionally, improvement of knee strength (kgf/kg) might be caused by body weight loss because of improvement of edema and body fat because it was calculated as the body weight ratio. All these limitations may affect the interpretation of the results in this study. Therefore, our findings require confirmation from multicenter studies, possibly using outcome measurements that minimize selection bias.

## 5 CONCLUSION

Successful CA for both PAF and Non‐PAF improved QOL and exercise tolerance. Additionally, CA improved leg strength in the Non‐PAF group but not in the PAF group. These findings suggest that CA may improve exercise tolerance through different mechanisms in PAF and Non‐PAF patients.

## FUNDING INFORMATION

This study received no financial support from commercial sources, and the authors have no conflicts of interest to declare.

## CONFLICT OF INTEREST STATEMENT

Authors declare no conflict of interests for this article.

## ETHICS STATEMENT

Not applicable.

## PATIENT CONSENT STATEMENT

Not applicable.

## Supporting information


Data S1:

## Data Availability

The data are available on the request to the corresponding author.
