# Supplementary material for: Different effects of catheter ablation on exercise tolerance, leg strength, and quality of life in paroxysmal versus persistent atrial fibrillation
Source: J Arrhythm. 2025 Jan 14;41(1):e13220. doi: 10.1002/joa3.13220 (PMC11730720; doi:10.1002/joa3.13220)
Supplement: Supplementary file 1 — Data S1: [file JOA3-41-e13220-s001.docx]

Supplemental Table 1.

Changes in exercise tolerance parameters and QOL questionnaire tests from baseline to follow-up.

|  | PAF | | |  | Non-PAF | | |
| --- | --- | --- | --- | --- | --- | --- | --- |
|  | Baseline | 3M  after CA | 6M  after CA |  | Baseline | 3M  after CA | 6M  after CA |
| **Exercise tolerance parameters** |  |  |  |  |  |  |  |
| 6MWD (m) | 486.6 ± 74.1 | 520.1 ± 84.1^＊^ | 527.1 ± 83.4^＊^ |  | 521.2 ± 86.8 | 530.1 ± 93.5 | 547.5 ± 94.9^＊^ |
| Maximal HR at 6MWD (bpm) | 113.8 ± 29.9 | 116.7 ± 19.7 | 117.5 ± 19.7 |  | 139.8 ± 29.9 | 114.9 ± 24.9^＊^ | 111.8 ± 26.3^＊^ |
| Mean grip strength (kg) | 32.6 ± 8.9 | 32.7 ± 8.8 | 32.6 ± 8.8 |  | 32.4 ± 10.2 | 32.6 ± 9.7 | 30.8 ± 10.4 |
| Mean knee extension force (kgf/kg) | 56.7 ± 12.2 | 57.2 ± 13.6 | 57.1 ± 13.6 |  | 54.9 ± 16.5 | 56.7 ± 15.2 | 58.4 ± 15.2^＊^ |
| Mean knee extension force (kg) | 37.7 ± 13.3 | 38.8 ± 13.4 | 38.2 ± 13.4 |  | 37.0 ± 11.9 | 37.8 ± 12.1 | 38.9 ± 12.2 |
| body weight (kg) | 65.3 ± 11.6 | 65.8 ± 11.7 | 66.1 ± 11.7 |  | 67.1 ± 11.3 | 66.3 ± 11.1^＊^ | 66.3 ± 11.2^＊^ |
| SMI [(kg)/(height (m)]^2^ | 7.0 ± 0.2 | 7.0 ± 1.4 | 7.0 ± 1.4 |  | 7.3 ± 0.2 | 7.3 ± 1.4 | 7.3 ± 1.4 |
| **QOL score** |  |  |  |  |  |  |  |
| AFQLQ score |  |  |  |  |  |  |  |
| Total score | 70.8 ± 17.8 | 88.9 ± 13.6^＊^ | 89.5 ± 13.6^＊^ |  | 77.3 ± 15.8 | 88.8 ± 13.1^＊^ | 90.5 ± 13.6^＊^ |
| AFQLQ1 | 15.0 ± 7.0 | 22.1 ± 5.4^＊^ | 22.3 ± 5.4^＊^ |  | 16.9 ± 6.6 | 21.9 ± 4.8^＊^ | 22.5 ± 4.8^＊^ |
| AFQLQ2 | 13.2 ± 4.6 | 16.9 ± 3.4^＊^ | 16.7 ± 3.4^＊^ |  | 14.2 ± 4.2 | 16.9 ± 3.5^＊^ | 16.6 ± 3.5^＊^ |
| AFQLQ3 | 42.6 ± 8.8 | 49.8 ± 7.5^＊^ | 50.6 ± 7.5^＊^ |  | 46.2 ± 7.2 | 50.0 ± 7.6^＊^ | 51.5 ± 7.6^＊^ |
| SARC-F (points) | 0.8 ± 1.1 | 0.5 ± 1.4 | 0.5 ± 1.4 |  | 0.8 ± 1.2 | 0.6 ± 1.4 | 0.3 ± 1.4^＊^ |
| 3Q score (points) | 4.0 ± 3.3 | 3.8 ± 3.4 | 4.1 ± 3.5 |  | 3.7 ± 3.4 | 5.0 ± 3.5^＊^ | 5.3 ± 3.7^＊^ |

Data presented as mean ± SD. * P < 0.05 vs. Compared with Baseline.

Abbreviations: refer to Tables 1 and 2

Supplemental Table 2.

Changes in Laboratory data and Echocardiographic parameters from baseline to follow-up.

|  | PAF | | |  | Non-PAF | | |
| --- | --- | --- | --- | --- | --- | --- | --- |
|  | Baseline | 6M after CA | *P value* |  | Baseline | 6M after CA | *P value* |
| **Laboratory data** |  |  |  |  |  |  |  |
| BNP (pg/mL) | 72.4 [34.0, 167.5] | 49.7 [25.8, 83.1] | 0.0541 |  | 168.0 [74.8, 218.8] | 39.4 [22.2, 81.2] | < 0.0001 |
| HANP (pg/mL) | 66.8 [37.5, 135.0] | 46.5 [24.9, 73.4] | 0.0006 |  | 103.0 [71.1, 136.5] | 44.0 [22.3, 67.6] | < 0.0001 |
| **Echocardiographic parameters** |  |  |  |  |  |  |  |
| LVEF (%) | 65.0 ± 7.9 | 64.1 ± 7.4 | 0.6780 |  | 63.8 ± 6.3 | 66.2 ± 3.5 | 0.0232 |
| LAD (mm) | 38.9 ± 5.5 | 36.0 ± 4.6 | 0.0003 |  | 41.1 ± 4.7 | 37.5 ± 5.3 | < 0.0001 |
| LVDD (mm) | 48.3 ± 5.9 | 46.9 ± 5.5 | 0.0572 |  | 47.1 ± 4.3 | 47.7 ± 4.0 | 0.1933 |
| E/e’ (mm) | 8.2 ± 2.8 | 7.7 ± 2.6 | 0.3542 |  | 8.2 ± 4.3 | 8.2 ± 2.0 | 0.4745 |

Data presented as the mean ± SD or the median with IQR.

Abbreviations: refer to Tables 1 and 2
